# Stagnant Stunting Rate despite Rapid Economic Growth—An Analysis of Cross Sectional Survey Data of Undernutrition among Children under Five in Papua New Guinea

**DOI:** 10.3934/publichealth.2016.1.25

**Published:** 2016-01-21

**Authors:** Xiaohui Hou

**Affiliations:** World Bank Group, 1818 H. St. NW

**Keywords:** nutrition, child health, economic growth, early child development, Papua New Guinea

## Abstract

**Background:**

Maternal and child under-nutrition is a pervasive and detrimental condition in Papua New Guinea (PNG). Despite the rapid economic growth during the past decade, the stunting rate for children under 5 has remained at 46 percent in PNG.

**Objective:**

The objective of the study was to analyze the association between the demographic, socioeconomic, and health-related factors with undernutrition for children less than five years old in PNG.

**Data and Method:**

The study used the 2009–2010 PNG Household Income and Expenditure Survey (HIES). The final sample included 3057 children under 5 years old. Logistic regression analyses were used to assess the odds of stunting, wasting and underweight. Odds ratios were adjusted for independent variables at household and individual levels. Statistical analyses were done with Stata (version 14).

**Findings:**

Logistic regression analysis showed household wealth was a significant predictor of stunting: children in the richest wealth quintile were 28.9% (0.711 [0.53–0.95]) less likely to be stunted than were children in the poorest wealth quintile. Other factors also associated with stunting include geographic locations, household head education and incidence of malaria. Similar results were found when wasting and underweight were dependent variables.

## 1. Introduction

In 2014, following a number of years of high growth, the Gross Domestic Product (GDP) per capita growth in Papua New Guinea (PNG) reached 8.0 percent. However, despite the relatively robust economic growth, maternal and child undernutrition remain pervasive and damaging conditions in PNG. Undernutrition, which encompasses stunting, wasting, underweight and deficiencies of micronutrients (essential vitamins and minerals), is one of the most significant causes of child mortality and morbidity[1]. Stunting in early life can have both short and long-term impacts on child and adult health. It is associated with poor cognition and educational performance in childhood. Stunting is also associated with low adult wages, lost productivity, and increased risk of nutrition-related chronic diseases when accompanied by excessive weight gain later in life [2].

The question arises as to why the general economic development in PNG has not led to improved nutritional outcomes. Economic development and poverty are key basic contributing factors for child malnutrition [3],[4]. UNICEF's conceptual framework of malnutrition [5] classifies the causes of malnutrition into three groups: immediate, underlying and basic causes. While dietary intake and health status are immediate causes of malnutrition; environmental, cultural and sociopolitical and economic contextual factors are all basic and underlying causes. General economic growth, measured by GDP per capita, predicts reduction in stunting rate in a reasonably large magnitude. One estimation suggested “a 10.0 percent increase in GDP per capita predicts a 5.9 percent reduction in stunting”, when economic growth was combined with other nutrition related interventions [6].

Despite the conceptual argument highlighting the strong linkages between economic growth and child stunting rate reduction, the empirical research has challenged this view. Vollmer et al. (2014) conclude that “the contribution of economic growth to the reduction in early childhood undernutrition in developing countries is very small, if it exists at all” [7]. This perspective was based on 121 Demographic and Health Surveys from 36 low-income and middle-income countries. Subramanyam et al. (2011) concluded that there was no consistent evidence that economic growth had contributed to reducing undernutrition in India using three waves of household level data [8].

Unfortunately, the research remains quite limited in PNG. The previous nutritional studies in PNG have reported low dietary intake of protein in the traditional diet [9]. Low energy and protein levels were also reported among children who lived in Lufa (Eastern Highlands Province) and Kaul (Madang Province) [10]. One study indicated moderate Iodine deficiency among a cohort of 350 school children (6–12 years old) in the Southern Highlands Province [11]. Semba et al. (2008) and Gibson (1999) showed that improving mothers' education could significantly reduce child malnutrition in Indonesia, Bangladesh and Papua New Guinea [12],[13]. Sharp et al. (1980) argued that malaria is a significant contributing factor to children's stunting status in Papua New Guinea's Highlands Region and the effect is most marked in children under two years of age [14]. However, in general, the incidence of malaria is lower in highland than lowland areas [15]. Bauze et al. (2012) showed the substantial within-province heterogeneity of under-five mortality and suggests that under-five mortality needs to be addressed at subnational levels [16]. Wand et al. (2012) highlighted the impact of geographical location on the risk factors, and its importance to understand both epidemiology and design interventions [17]. Another two papers showed the importance of the supplement zinc on children's stunting status using Indonesia and Papua New Guinea data respectively [18],[19].

The objective of this paper was to use the latest national representative survey, PNG Household Income and Expenditure Survey (HIES) 2009–2010, to further shed light on distribution of undernutrition and factors which are significantly associated with undernutrition in PNG to guide nutrition-related policies. This will contribute to the literature and policy debate on how to further address under-nutrition issues in PNG given that the general economic development has not led to improved nutritional outcomes.

## 2. Data and Methods

### 2.1. Data

The analysis in this paper uses the data from the 2009–2010 Papua New Guinea Household Income and Expenditure Survey (2009–2010 PNG HIES). The 2009–2010 HIES was the first comprehensive and nationally representative survey of the socioeconomic status of PNG households since the 1996 Household Survey of PNG. The 2009–2010 PNG HIES contains the final cross-section sample of 4,191 households. A comprehensive set of multi-topic questionnaires was designed to elicit information on key topics such as family demography, education, health, employment, and consumption. The survey also collected information on height, weight and date of birth for children under five. The final sample contains 3,056 children under 5 years old. The data are nationally representative at the regional level.

This study used the World Health Organization Child Growth Standards for the classification of stunting, wasting and underweight status [20]. Children are classified as stunted, wasted and underweight if their height-to-age Z-score, weight-to-height Z-score and weight-to-age Z-score are below -2 respectively. Children are classified as severe stunted, severe wasted and severe underweight if their height-to-age Z-score, weight-to-height Z-score and weight-to-age Z-score are below -3 respectively [20].

Data from two parts of the survey were combined to estimate dietary intake: the household stocks data and the personal diary data. The household stocks of food data, involved an interviewer asking the household head or representative about food currently in the house on day 1 and day 14 of the survey. The stocks were weighed by the interviewer. Nineteen food items, identified as the nineteen most common items in the 1996 HIES, were specified on the questionnaire. Other items were found, weighed and listed. The second source of data was derived from the participant's recorded transactions in a personal diary for 14 days. A total number of 295,804 transactions involving food and alcohol were extracted from this diary.

The quantity of food was converted to calories using food composition information from the New Zealand FOODfiles 2012 database [21]. Energy and nutrient composition for each item was estimated by taking an average of relevant items from the FOODfiles database. The difference between stock levels on day1 and day14, plus additional purchased foods were converted to the total calories consumed by the households.

Total calories consumed by households during the two weeks were then converted to calories per capita (adult equivalent) per day. Per capita is defined by adult equivalent using children 0–14 as 0.5 adult. Guests who regularly have meals in the households were added while members of households who do not regularly eat at home were deducted. Since per adult equivalent calories were strongly right skewed, this was converted into quintiles to be included in the regression. Per adult equivalent protein intake was used as the proxy for quantity and quality of diet. Adequate protein intake has been a concern and controversy in international nutrition community in the past 50 years [22]–[24]. In PNG, the most source of proteins are from meat and offal, fish and sea food, milk and eggs, etc [25].

### 2.2. Data Analysis

Two types of analyses were implemented. The first was a preliminary analysis using exploratory data technique to examine univariate and bivariate relationship. Histogram charts or bivariate correlation charts were presented to show the distribution and association of undernutrition variables with key independent variables. The second, and more comprehensive analysis, utilized a logistic regression. The dependent variables are children's undernutrition status including stunting, wasting, and underweight [26]. Covariates related to household include household wealth quintiles, measured by per capita household expenditures; per adult equivalent protein intake; and household head education level, as a proxy for household overall knowledge on nutritional intake. PNG regional variations were controlled as the food availability and diet habits are quite different across the regions. Covariates at children level are age (months) and gender. Children having had diarrhea and malaria in the past 30 days were also controlled because of the association between morbidity and undernutrition [27].

## 3. Results

### 3.1. Overall Prevalence

**Figure 1.**
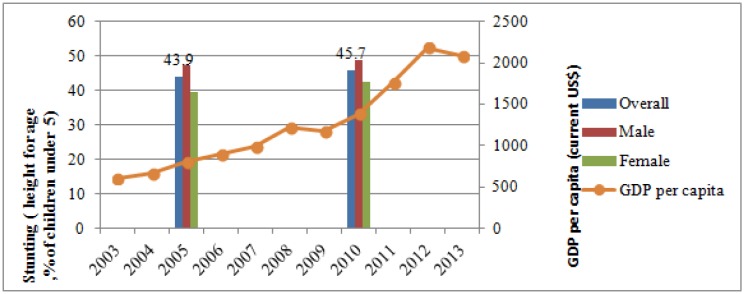
Stunting Rate for Children Under 5 and GDP per Capita Growth from 2005 to 2010. Data source: GDP is from World Development Indicators; stunting rate in 2010 is from Household Income and Expenditure Survey (HIES) 2009–2010; 2005 stunting rate is from 2005 National Nutrition Survey.

**Figure 2.**
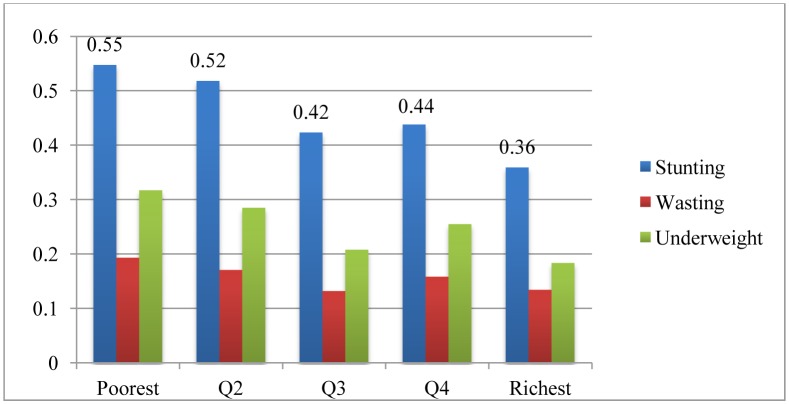
The Stunting, Underweight and Wasting Across Wealth Quintile in PNG. Data source: HIES 2009–2010

The stunting rate, indicating chronic restriction of a child's potential growth for children under 5 in PNG, was estimated at 46 percent in 2010. Figure 1 showed that in PNG despite GDP growth of 6.85 percent on average from 2005 to 2010, the stunting rate remained basically stagnant from the 44 percent in 2005. The poorest quintile had the highest stunting rate at 55 percent. However, the stunting rate among the richest quintile was also quite high (36 percent), and similarly high among the third and fourth quintiles. In terms of underweight and wasting, the fourth quintile had even worse nutritional outcomes compared with the third quintile (Figure 2).

Undernutrition in PNG is prevalent and severe, and varies across the regions. According to the HIES 2010 data, the overall stunting, underweight, and wasting rates were high, 46 percent, 25 percent and 15.8 percent, respectively. Not only were the overall rates high; the severe cases among the stunted, underweight and wasted children were also high at around 50 percent in each category (Table 1).

The incidence of stunting was not evenly distributed across the regions (Table 1). The stunting rate was high across all the regions but extremely high at 61.5 percent in the Highlands Region. The Islands Region had the lowest stunting rate at 38.1 percent. Contrary to the distribution of stunting rate, wasting rate and underweight rate were the highest among the Islands Region at 29.2 percent. This was similar to the earlier findings using the HIES 1996 [28].

**Table 1.** Summary statistics of dependent variables and independent variables.

Variables	Overall		Southern		Highlands		Momase		Islands	
	Mean		Mean		Mean		Mean		Mean	
Stunting (z-score < -2)	45.71%		37.75%		61.48%		47.55%		38.10%	
Wasting (z-score < -2)	15.76%		17.71%		9.85%		16.02%		18.85%	
Underweight (z-score < -2)	24.97%		22.95%		22.31%		26.77%		29.22%	
Severe stunting (z-score < -3)	21.43%		17.69%		29.70%		23.04%		15.15%	
Severe wasting (z-score < -3)	8.83%		8.90%		6.51%		8.28%		12.86%	
Severe underweight (z-score < -3)	9.13%		7.72%		9.15%		10.10%		10.17%	
Per capita expenditure quintile 1	20.02%		8.00%		24.24%		28.96%		22.08%	
Per capita expenditure quintile 2	19.99%		15.22%		25.68%		19.58%		23.59%	
Per capita expenditure quintile 3	20.02%		21.44%		23.43%		16.88%		18.83%	
Per capita expenditure quintile 4	20.02%		23.42%		16.05%		18.85%		20.35%	
Per capita expenditure quintile 5	19.95%		31.92%		10.59%		15.73%		15.15%	
Per capita protein intake quintile 1	20.06%		18.37%		11.09%		27.73%		20.09%	
Per capita protein intake quintile 2	19.96%		14.65%		28.16%		18.37%		24.11%	
Per capita protein intake quintile 3	20.06%		18.06%		24.23%		18.15%		22.93%	
Per capita protein intake quintile 4	19.92%		22.81%		20.48%		17.26%		18.20%	
Per capita protein intake quintile 5	19.99%		26.11%		16.04%		18.49%		14.66%	
Household head's education: no formal education	27.41%		17.98%		41.73%		29.06%		25.32%	
Household head's education: primary	28.30%		25.00%		25.20%		34.06%		27.71%	
Household head's education: above primary	44.29%		57.02%		33.07%		36.88%		46.97%	
Urban	46.16%		67.49%		20.22%		46.98%		32.68%	
Male	52.40%		51.48%		53.29%		53.33%		51.30%	
Children had diarrhea in the last 30 days	5.56%		3.85%		7.70%		7.40%		2.60%	
Children had malaria in the last 30 days	7.10%		7.02%		2.25%		7.29%		13.42%	
Age in month	29.30	(17.02)	29.04	(17.40)	29.93	(16.19)	29.13	(17.07)	29.40	(17.17)
Sample size	3057		1012		623		960		462	

Note: The quintiles for both per adult equivalent protein intake and expenditure were presented in the table due to large variations across the regions.

### 3.2. Malnutrition and Age

**Figure 3.**
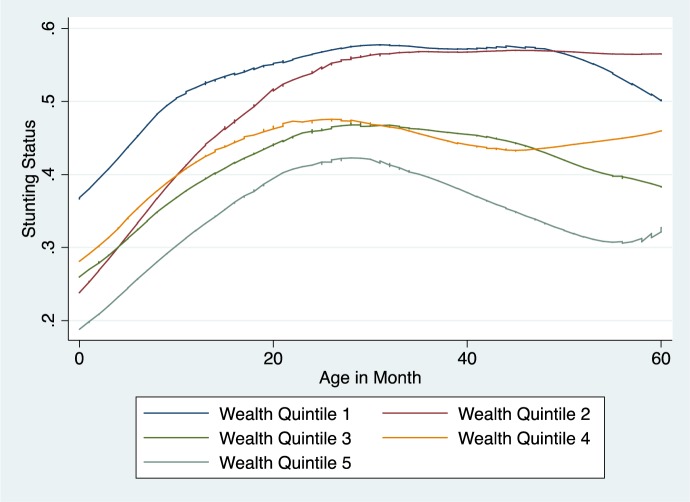
The Rate of Stunting Rate and Age Across Five Wealth Quintiles Data source: HIES 2009–2010

A closer examination of stunting and age by wealth quintiles showed that the stunting rate sharply rose in the first 24 months (Figure 3). The trend for the first 24 months was similar across the five quintiles while the levels were different. Children in lowest wealth quintile had the highest stunting rate, followed by children in the second lowest wealth quintile. Children in the richest quintile had the lowest stunting rate. However, there was a mild bend in the curve for the richest quintiles while the stunting rate plateaued for the poor quintiles.

### 3.3. Caloric and Protein Intake according to economic status

Caloric and protein intake increased with economic status. Economic status was measured by log per capita expenditure. Log per adult equivalent calories intake increased with the increase in log per capita expenditure; same for log per adult equivalent protein intake. This implied that both food quality and quantity improved with the increase of economic status. This also indicated that log per adult equivalent calories and log per adult equivalent protein intake were co-linear thus only caloric intake was included in the regression.

### 3.4. Results from Logistic Regression

**Figure 4.**
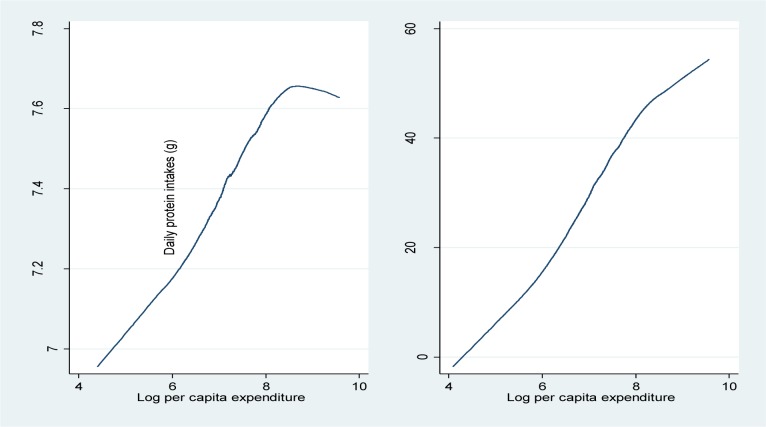
Log per Capita Expenditure and Log Daily Calorie and Daily Protein Intakes. Data source: HIES 2009–2010

Table 2 shows the logistic analysis of associated factors to stunting, wasting and underweight for children under age five. Odds ratios were presented in the table. Household wealth was a significant indicator for children's stunting status. Children in the richest wealth quintile were 28.9% (0.711 [0.53–0.95]) less likely to be stunted, 33.8% (0.662[0.445–0.983]) less likely to be wasted, and 32% (0.680[0.488–0.948]) less likely to be underweighted than were children in the poorest wealth quintile. Per adult equivalent caloric intake quintiles were not significant when per capita expenditure was controlled in the regression; however, when per capita expenditure was not controlled, children in the highest quintile of per adult equivalent caloric intake were 24% (0.760[0.607–0.952]) less likely to be stunted. This reflects the co-linear relationship between per capita expenditure and per adult equivalent caloric intake results presented earlier in Figure 4.

Household head education level had a significant effect on children's stunting status after controlling for other covariates. Children with household heads having above primary education level were 20.4% (0.796 [0.634–0.999] less likely to be stunted, 34.1% (0.659[0.490–0.886]) less likely to be wasted and 41.3% (0.587 [0.461–0.747]) less likely to be underweight, compared with children with household heads having no formal education after controlling for per capita expenditure quintiles, per adult equivalent caloric intake quintiles and other key covariates.

Other findings showed that urban children were less likely to be stunted, and boys were more likely to be stunted. Geographic locations were crucial factors that contribute to children's stunting. Compared with children in Southern region, children in Highlands region were 2.055([1.643–2.570]) times more likely to be stunted; children in Momase region were 1.277([1.057–1.543]) times more likely to be stunted; and children in Islands region were 18.2% (0.818[0.644–1.038]) less likely to be stunted. Further, children's health status, more specifically, having had malaria in the past 30 days, had a significant correlation with child stunting and underweight.

**Table 2.** Logistic Regression results of multivariate relationship of stunting, wasting, and underweight with independent variables (Odds ratio and 95% confidence intervals are presented, N = 3057)

	Dependent Variables					
	Stunting		Wasting		Underweight	
Independent Variables						
Wealth quintile 2	0.980		0.876		0.964	
	(0.772 – 1.244)		(0.642 – 1.196)		(0.745 – 1.248)	
Wealth quintile 3	0.734**		0.648**		0.685**	
	(0.569 – 0.946)		(0.457 – 0.920)		(0.514 – 0.915)	
Wealth quintile 4	0.899		0.788		0.979	
	(0.687 – 1.177)		(0.549 – 1.130)		(0.727 – 1.319)	
Wealth quintile 5	0.711**		0.662**		0.680**	
	(0.533 – 0.950)		(0.445 – 0.983)		(0.488 – 0.948)	
Calorie quintile 2	0.967	0.923	1.109	1.036	1.038	0.988
	(0.767 – 1.220)	(0.736 – 1.158)	(0.813 – 1.513)	(0.764 – 1.404)	(0.801 – 1.346)	(0.767 – 1.274)
Calorie quintile 3	0.944	0.866	1.104	0.985	0.951	0.870
	(0.745 – 1.198)	(0.691 – 1.087)	(0.799 – 1.525)	(0.723 – 1.343)	(0.725 – 1.249)	(0.670 – 1.128)
Calorie quintile 4	0.861	0.785**	1.102	0.973	0.897	0.823
	(0.677 – 1.095)	(0.627 – 0.983)	(0.797 – 1.523)	(0.719 – 1.317)	(0.682 – 1.180)	(0.637 – 1.063)
Calorie quintile 5	0.853	0.760**	1.071	0.921	1.107	0.986
	(0.668 – 1.089)	(0.607 – 0.952)	(0.766 – 1.497)	(0.677 – 1.254)	(0.842 – 1.456)	(0.767 – 1.267)
Highlands	2.055***	2.101***	0.408***	0.418***	0.674***	0.692***
	(1.643 – 2.570)	(1.682 – 2.625)	(0.290 – 0.573)	(0.298 – 0.586)	(0.518 – 0.877)	(0.532 – 0.900)
Momase	1.277**	1.339***	0.771**	0.819	1.001	1.054
	(1.057 – 1.543)	(1.112 – 1.611)	(0.599 – 0.993)	(0.639 – 1.048)	(0.806 – 1.243)	(0.852 – 1.303)
Islands	0.818*	0.839	0.975	0.996	1.093	1.125
	(0.644 – 1.038)	(0.662 – 1.065)	(0.721 – 1.318)	(0.738 – 1.346)	(0.841 – 1.421)	(0.867 – 1.461)
Urban	0.733***	0.675***	0.942	0.845	0.702***	0.652***
	(0.612 – 0.879)	(0.574 – 0.793)	(0.733 – 1.209)	(0.677 – 1.055)	(0.570 – 0.865)	(0.541 – 0.786)
Household head education (primary)	0.866	0.857	1.011	0.991	0.868	0.861
	(0.653 – 1.147)	(0.647 – 1.134)	(0.710 – 1.440)	(0.697 – 1.410)	(0.647 – 1.166)	(0.642 – 1.154)
Household head education (above primary)	0.796**	0.768**	0.659***	0.626***	0.587***	0.567***
	(0.634 – 0.999)	(0.613 – 0.961)	(0.490 – 0.886)	(0.468 – 0.838)	(0.461 – 0.747)	(0.446 – 0.719)
Male	1.300***	1.302***	1.108	1.108	1.189**	1.193**
	(1.122 – 1.507)	(1.124 – 1.508)	(0.907 – 1.355)	(0.907 – 1.354)	(1.005 – 1.407)	(1.009 – 1.411)
Children had diarrhea in the past 30 days	1.152	1.130	0.857	0.836	1.043	1.023
	(0.834 – 1.592)	(0.819 – 1.560)	(0.540 – 1.359)	(0.528 – 1.325)	(0.725 – 1.501)	(0.712 – 1.470)
Children had malaria in the past 30 days	1.335**	1.305*	0.939	0.919	1.351*	1.311*
	(1.003 – 1.775)	(0.982 – 1.734)	(0.636 – 1.384)	(0.624 – 1.354)	(0.993 – 1.836)	(0.966 – 1.781)
Age in month	1.007***	1.007***	0.989***	0.989***	1.007***	1.006**
	(1.003 – 1.012)	(1.003 – 1.012)	(0.984 – 0.995)	(0.983 – 0.995)	(1.002 – 1.012)	(1.001 – 1.011)

Note: wealth quintiles were measured by per capita expenditure and lowest wealth quintile was the comparison group; caloric intake quintiles were measure by per adult equivalent protein intake and protein intake quintile 1 was the comparison group; Southern region, household head with no formal education were the comparison group in the logistics regression. Odds Ratio and 95% confidence intervals (in parenthesis) are presented in the table. *** *p* < 0.01, ** *p* < 0.05, * *p* < 0.1

## 4. Limitations

Due to data limitation, this paper could not establish the causal links between various factors and nutrition outcomes, nor answer why general economic development had not improved the nutrition outcomes in PNG. Rather, it analyzed the association between the demographic, economic, geographic and health-related factors on nutritional status at household and individual level. Distribution of caloric and nutrition intake within households (especially protein and micronutrient rich foods) is unequal and does not favor children [29]. However, the analyses did not have individual level based nutritional intake information thus used per adult equivalent caloric intake quintiles as independent variables.

## 5. Discussion

The paper analyzed the association between the demographic, socioeconomic, and health-related factors on nutritional status for children under five. The stunting rate sharply rose in the first 24 months. The logistic regression results showed family wealth was a significant factor associated with the stunting rate. In addition, caloric intake and the history of incidence of malaria were highly correlated with the likelihood of stunting.

Growth faltering often begins in utero and continues for the first two years of life [7]. The main window of opportunity to prevent stunting is the intra-uterine and postnatal periods, from conception until 24 months. Significant reductions in stunting can be achieved through a comprehensive set of priority interventions during this critical development stage. The recommended feeding of children is exclusive breastfeeding for the first 6 months of life and continued breastfeeding through the second year of life. In PNG, however, infants are introduced to solid food at a much earlier stage. One study examined various data sources in PNG and showed that exclusive breastfeeding rates were low: about 80% at 1 month; 36% at 4–5 months; and almost 10% of neonates and 27% of infants were given semi-solid or solid food before 4 months of age [30]. This suboptimal breastfeeding practice for young children increases not only the incidence of both stunting and wasting, but also the mortality and morbidity of children.

The results showed that even in the better-off quintiles, children's stunting rate remained high. The evidence suggested that women lacked knowledge of quality feeding for their children even among better-off households. The reasons for suboptimal feeding were multi-layered. Some were culturally linked; some were due to lack of understanding of the consequences of suboptimal feeding; and some were due to the lack of quality supplementary food. The evidence showed due to the cultural custom that men were not allowed having sex with breastfeeding women, husbands sometimes would have stopped mother from breastfeeding [31]. The same study also showed that more than one third of mothers surveyed did not give colostrum to babies with cultural beliefs that colostrum may harm the baby. Colostrum was described as “dirty”, “unclean”, “contained infected pus”, “waste from mother's body”, “not food for the baby”, or “infectious to a child and can cause yellow eyes” [31]. Rural mothers' knowledge about the benefits of breastfeeding and in particular about the importance of exclusive breastfeeding in a child's first six months of life seems extremely poor.

Children living in homes where the heads of the households have a higher level of education were less likely to be malnourished after controlling for wealth information. A study found that lack of access to information on proper nutrition was an important factor contributing to the high levels of malnutrition in PNG [32]. This is particularly the case in remote rural communities with prevalent illiteracy and lack of access to education. Stand-alone nutrition education programs have been carried out in the Pacific region [33], and their effectiveness depends on the intensity and quality of implementation. This includes appropriate communication strategies (for example, combination of mass media and interpersonal) and targeting the right audience. Good formative research is needed to identify key obstacles, identify who influences mothers' decisions in adopting healthy behavior (i.e. grandmother, husband, community leaders) and ensure that nutrition education reaches these groups. A wider body of evidence suggests that nutrition education programs can be more effective when combined with other interventions such as to increase food or income availability or to provide nutritional supplements [32], [34],[35]. Agricultural interventions that aim to improve income and productivity tend to be successful in improving the nutrition status of children when they include a nutrition education component [36].

Health workers have critical roles in addressing undernutrition in PNG. First, diseases, particularly infectious diseases, are important determinants of stunting [6]. Diarrhea is well documented in the literature as a contributor to stunting because of its association with malabsorption of nutrients [37],[38]. The literature also suggests malaria is an important determinant of stunting [39]. In PNG, the regression result did not show significant association between diarrhea and malnutrition. However, in PNG children having malaria in the past 30 days were significantly more likely to be stunted. It is hard to determine the causal effects of malaria and stunting. Malaria affects children's ability to absorb nutrition due to the malfunction of red blood cells after the incidence of malaria, which then has a negative effect on children's height and weight. At the same time, children with malnutrition may be at a higher risk of getting malaria because of their overall living environment and access to mosquito nets.

Second, stunting often goes unrecognized because short stature is so common in some communities in PNG, particularly in rural highlands [40]. Thus, regular preventive programs such as Growth Monitoring and Promotion are necessary [41]. Measuring length (up to 24 months) or height (from 24 months onwards) should become standard practice when assessing a child either through postnatal visits or outreach visits conducted by health workers [41]. Health workers should be trained in delivering messages on how to improve nutrition outcomes. Such trainings should also be broadened to teachers, social workers and extension officers (locally known as didimen and didimeries). Supplies of ready-to-use therapeutic foods should be available in health facilities where (acute) malnutrition cases are more prevalent. These could be included in the medical kits distributed to health centers, in which capacity of treatment to the Moderate Acute Malnutrition and Severe Acute Malnutrition is extremely limited. Building human resource capacity to address undernutrition issues is an imperative need in the country. This requires strategy, financing and leadership's commitment.

## 6. Further Agenda

While PNG continues to battle a high stunting rate, it also faces emerging non-communicable diseases. As evidence has shown, an adult is more likely to develop non-communicable diseases if he/she was stunted as a child [2]. The body's ability to metabolize food is being programmed in the first 1000 days from conception and disruption to this process contributes to obesity and diabetes later in life [37]. This will pose a huge financial burden not only to families, but also to the public health financing schemes.

Further analytics are required to better understand the causes for malnutrition and the effects of various interventions. The quality of HIES data collection methods, particularly the questionnaire design, can be further strengthened to make it a more useful tool in identifying areas for health policy interventions and reform. To the extent possible, regular nutrition surveys to monitor nutrition status add tremendous value to understand the progress of improving nutrition outcomes and the causal links. Additional analytical work that can further shed light on combating the undernutrition issue includes: investigating the prevalence of deficiencies of micronutrients (vitamin A, zinc, iron, and iodine) in the subnational population; obtaining a better understanding of the nutrition inventions to date; and analysis of the lessons learned in PNG. These efforts will help in scaling up effective interventions using various platforms and linking child nutrition with cognitive development for school readiness and beyond.

To move toward more committed actions, it is critical to improve the political environment, aligning multiple actors, and advancing policies and legislation. The government has made some progress. The National Nutrition Policy was recently endorsed by a number of departments in addition to the National Department of Health in early 2015, highlighting the political commitment of multisectoral interventions to improve nutritional outcomes in the country. However, a much stronger political commitment along with adequate financing are required to ensure the successful implementation of the policy. Monitoring and evaluation of nutritional programs are also necessary to gather the latest evidence on the effective and efficient ways to improve nutrition across different regions in PNG.
